# Supplementary figures and images for: The validity evaluation of different 16srRNA gene primers for helicobacter detection urgently requesting to design new specific primers
Source: Sci Rep. 2022 Jun 24;12:10737. doi: 10.1038/s41598-022-14600-4 (PMC9232570; doi:10.1038/s41598-022-14600-4)

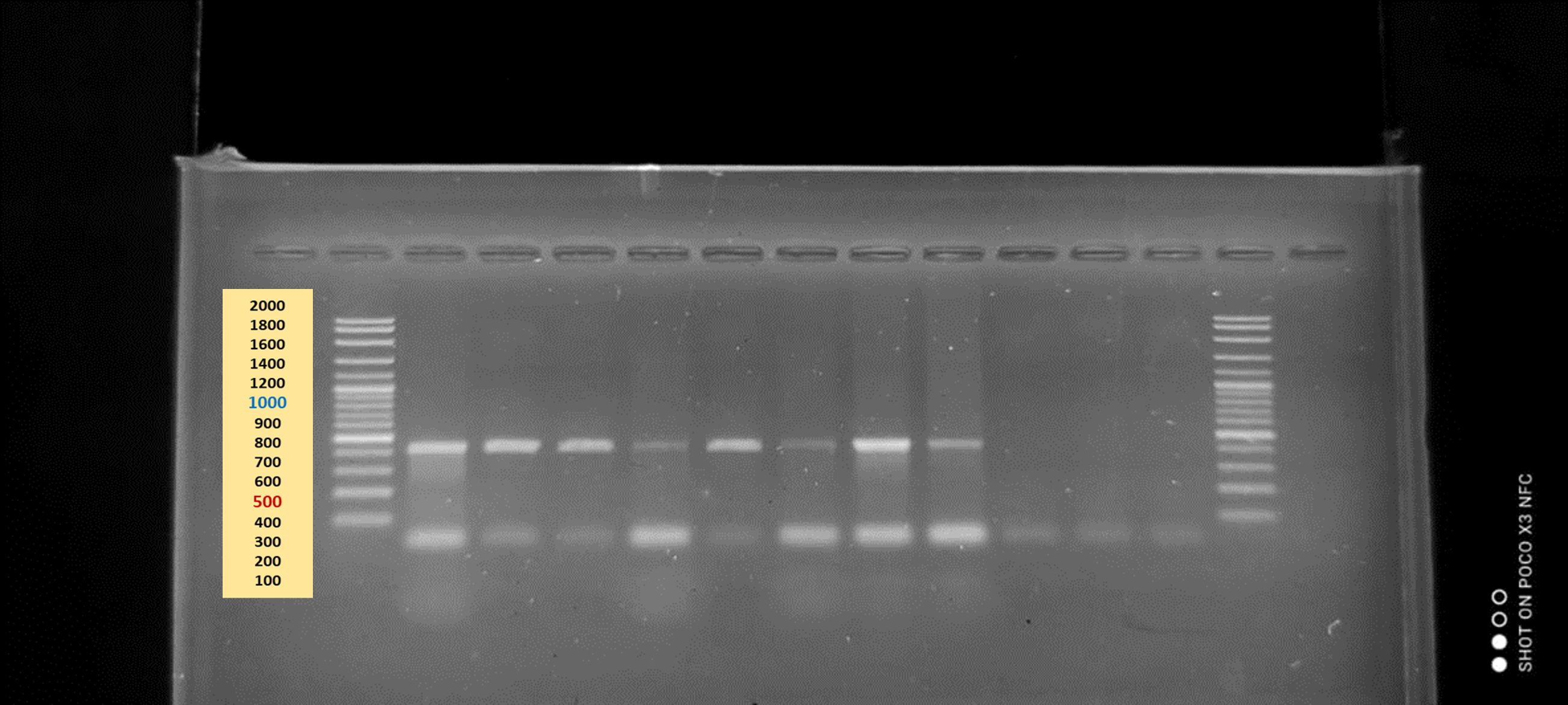

Supplement: Supplementary file 1 — Supplementary Information 1. [file 41598_2022_14600_MOESM1_ESM.png]

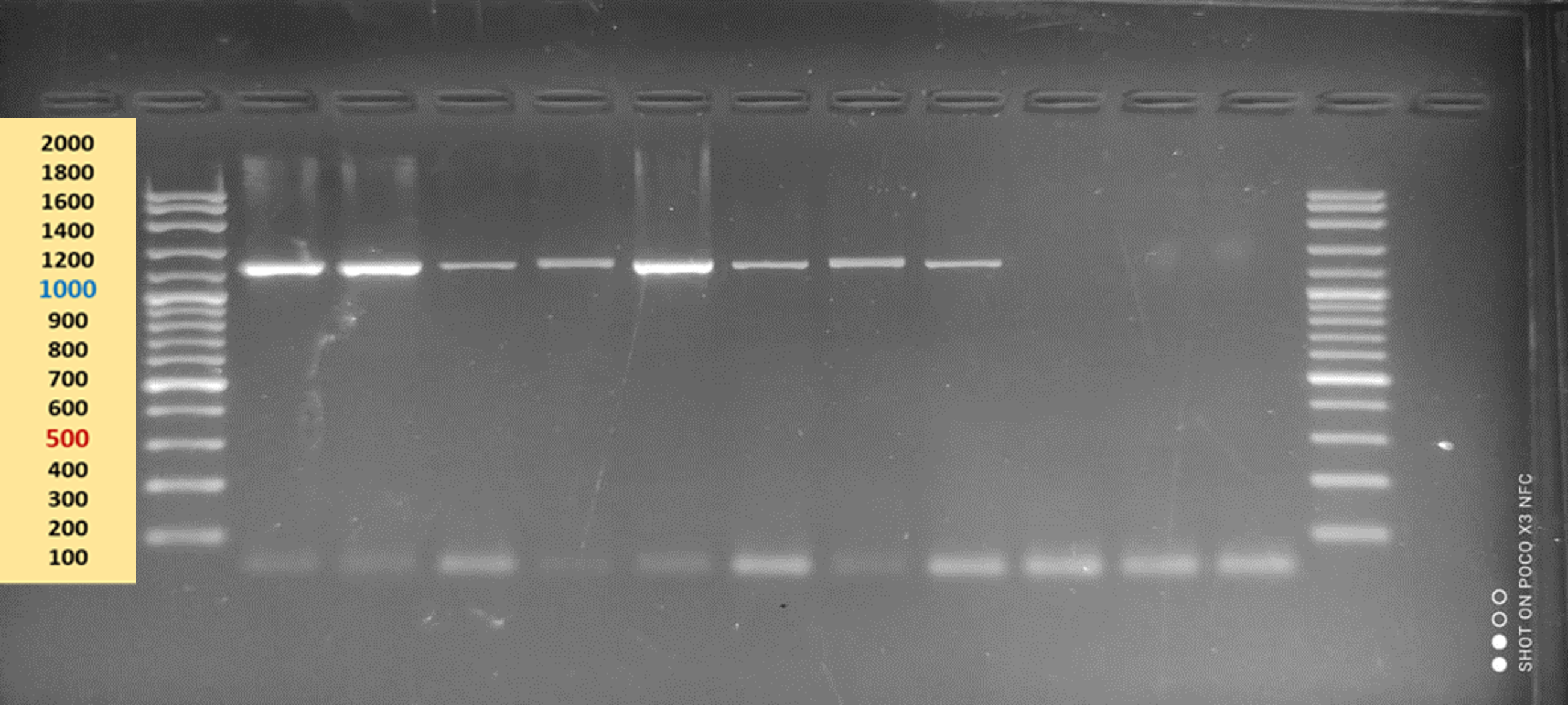

Supplement: Supplementary file 2 — Supplementary Information 2. [file 41598_2022_14600_MOESM2_ESM.png]

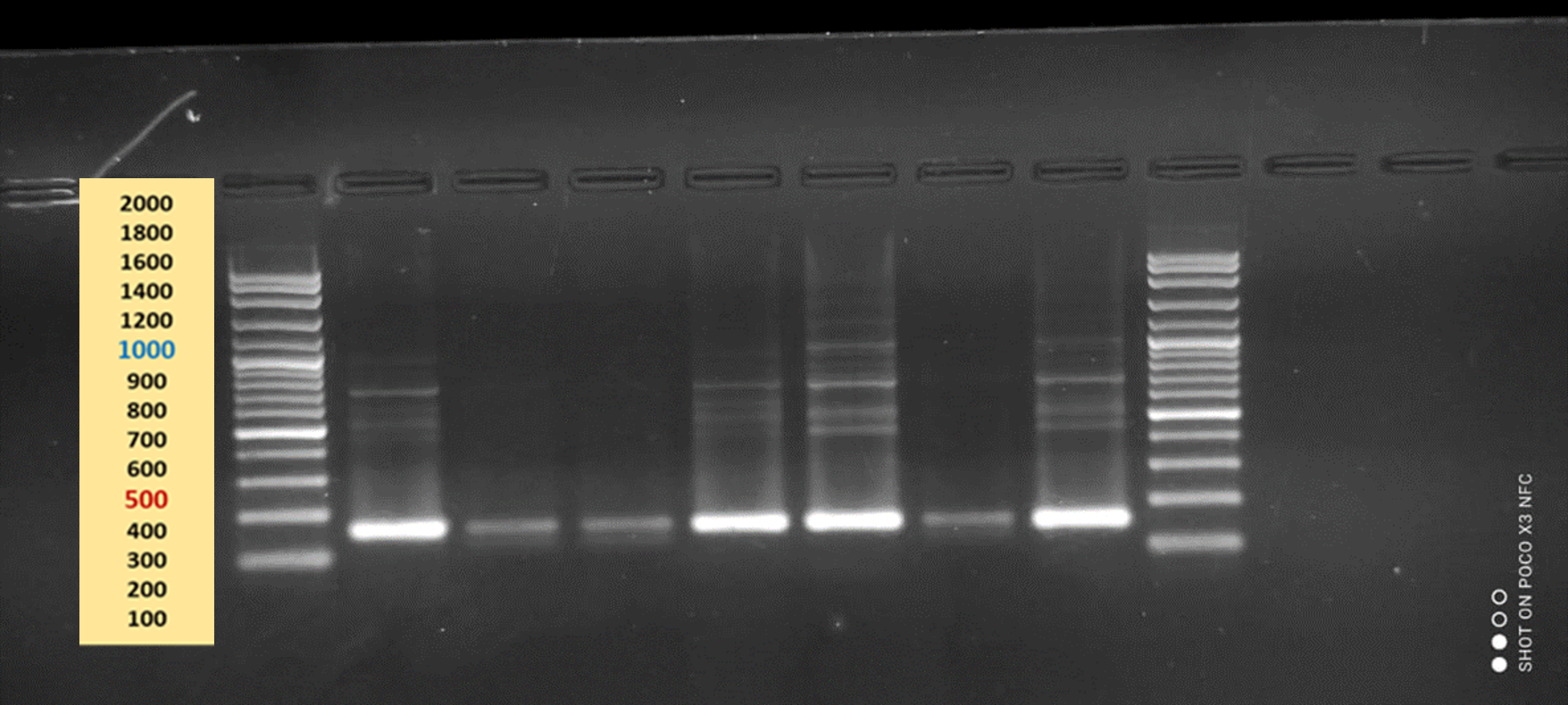

Supplement: Supplementary file 3 — Supplementary Information 3. [file 41598_2022_14600_MOESM3_ESM.png]

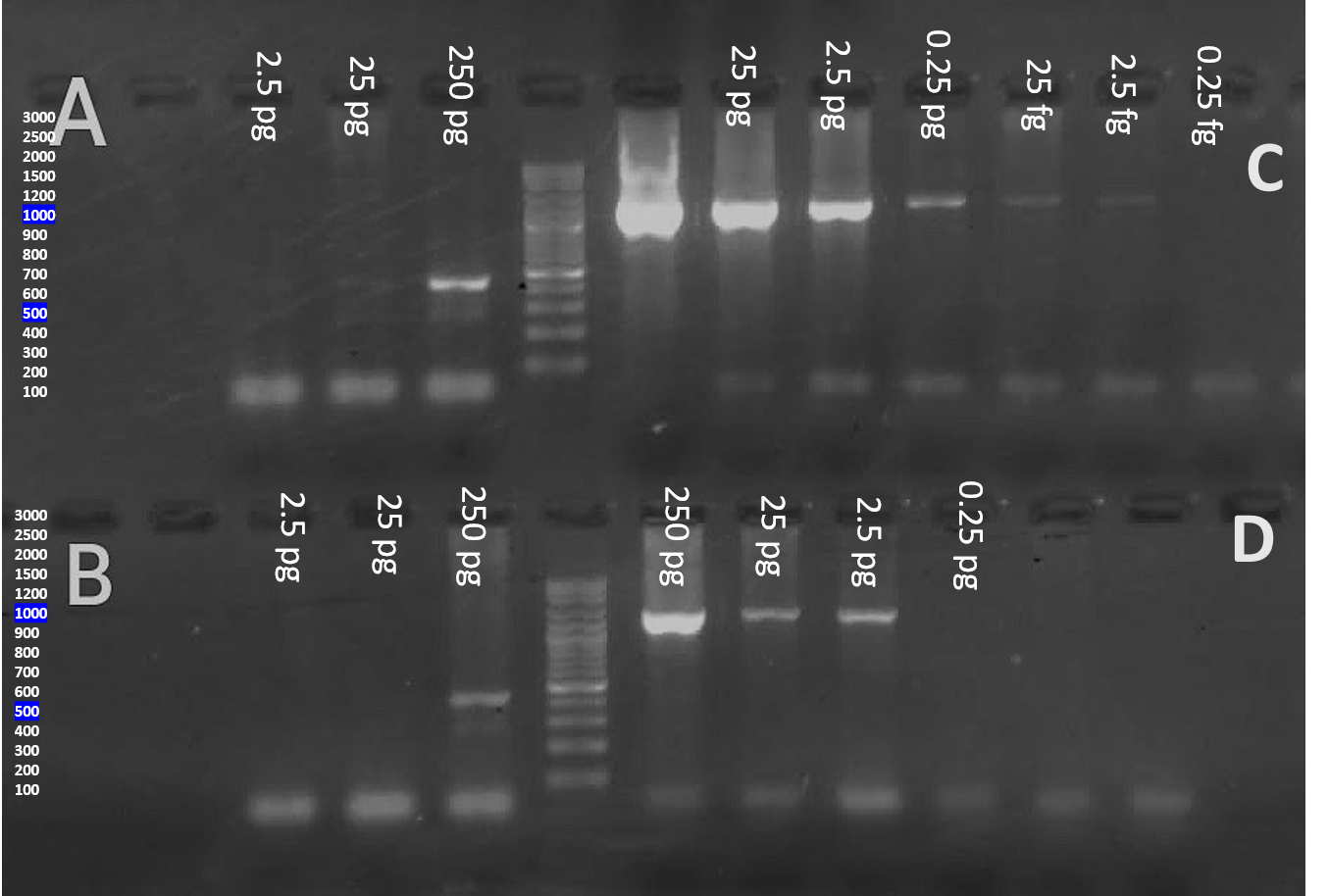

Supplement: Supplementary file 4 — Supplementary Information 4. [file 41598_2022_14600_MOESM4_ESM.png]
