# Supplementary material for: The validity evaluation of different 16srRNA gene primers for helicobacter detection urgently requesting to design new specific primers
Source: Sci Rep. 2022 Jun 24;12:10737. doi: 10.1038/s41598-022-14600-4 (PMC9232570; doi:10.1038/s41598-022-14600-4)
Supplement: Supplementary file 6 — Supplementary Information 6. [file 41598_2022_14600_MOESM6_ESM.docx]

**Results**

**Table 1. Results of screening primers** **in detection of 62 *Helicobacter* species (in silico no mismatch) and 62 *Helicobacter* species (in silico two mismatch)**

| **Screening Primers** | **In Silico no mismatch** | | **In Silico two mismatch** | |
| --- | --- | --- | --- | --- |
|  | **Positive** | **Negative** | **Positive** | **Negative** |
| **CONS1** | 55 (88.7%) | 7 (11.3%) | 60 (96.8%) | 2 (3.2%) |
| **Nested A + AN** | 58 (93.5%) | 4 (6.5%) | 61 (98.4%) | 1 (1.6%) |
| **H276** | 60 (96.8%) | 2 (3.2%) | 60 (96.8%) | 2 (3.2%) |
| **Outer + Inner** | 58 (93.5%) | 4 (6.5%) | 60 (96.8%) | 2 (3.2%) |
| **Heli-nestS + Heli** | 5 (8.1%) | 57 (91.9%) | 6 (9.7%) | 56 (90.3%) |
| **Hcom1** | 1 (1.6%) | 61 (98.4%) | 61 (98.4%) | 1 (1.6%) |
| **16S rRNA-F*** | 0 (0.0%) | 62 (100%) | 1 (1.6%) | 61 (98.4%) |
| **16S rRNA*** | 0 (0.0%) | 62 (100%) | 0 (0.0%) | 62 (100%) |
| **hspG*** | 0 (0.0%) | 62 (100%) | 0 (0.0%) | 62 (100%) |
| **BFHpyl*** | 0 (0.0%) | 62 (100%) | 1 (1.6%) | 61 (98.4%) |
| **Heid*** | 0 (0.0%) | 62 (100%) | 0 (0.0%) | 62 (100%) |

* 16S rRNA-F, 16S rRNA, hspG, BFHpyl and Heid primers tested negative for detection of all 62 Helicobacter species (in silico no mismatch), whereas 16S rRNA, hspG and Heid primers tested negative in detection of all 62 Helicobacter species (in silico two mismatch). 16S rRNA-F and BFHpyl primers tested negative in detection of 61 Helicobacter species (in silico two mismatch).

**Table 2. Screening primers test** **results for detection of 62 *Helicobacter* strains (no mismatch)**

| **Gold standard primer** | **Screening Primers** | **SEN (%)** | **SPC (%)** | **PPV (%)** | **NPV (%)** | **FPR (%)** | **FNR (%)** | **Acc (%)** | **BA (%)** | **LR+** | **LR-** | **DOR**  **(LR+ / LR-)** | ***X^2^*** | ***Kappa* Value** | ***P* value** |
| --- | --- | --- | --- | --- | --- | --- | --- | --- | --- | --- | --- | --- | --- | --- | --- |
| **CONS1** | **Nested A + AN** | 100 | 57.1 | 94.8 | 100 | 42.9 | 0.0 | 95.2 | 78.6 | 2.33 | 0.000 | - | 24.790 | 0.703 | 0.0001 |
|  | **H276** | 98.2 | 14.3 | 90.0 | 50.0 | 85.7 | 1.8 | 88.7 | 56.2 | 1.15 | 0.127 | 9 | 0.388 | 0.181 | 0.533 |
|  | **Outer + Inner** | 98.2 | 42.9 | 93.1 | 75 | 57.1 | 1.8 | 91.9 | 70.5 | 1.72 | 0.042 | 40.5 | 11.20 | 0.505 | 0.001 |
|  | **Heli-nestS + Heli** | 7.3 | 85.7 | 80 | 10.5 | 14.3 | 92.7 | 16.1 | 64.5 | 0.509 | 1.08 | 0.47 | 0.0001 | -0.017 | 1 |
|  | **Hcom1** | 0.0 | 85.7 | 0.0 | 9.8 | 14.3 | 100 | 9.7 | 42.9 | 0.00 | 1.17 | 0.00 | 1.52 | -0.033 | 0.218 |

SEN: sensitivity; SPC: specificity; PPV: Positive predictive value; NPN: Negative predictive value; FPR: False positive rate; FNR: False negative rate; ACC: Accuracy; BA: Balanced accuracy; LR+: Positive likelihood ratio; LR-: Negative likelihood ratio; DOR: Diagnostic odds ratio; *X^2^*: Chi-Square (Continuity Correction).

16S rRNA-F, 16S rRNA, hspG, BFHpyl and Heid primers tested negative for detection of all 62 Helicobacter species (in silico no mismatch).

**Table 3. Screening primers test results for detection of 62 *Helicobacter* strains (two mismatch)**

| **Gold standard Primer** | **Screening Primers** | **SEN (%)** | **SPC (%)** | **PPV (%)** | **NPV (%)** | **FPR (%)** | **FNR (%)** | **Acc (%)** | **BA (%)** | **LR+** | **LR-** | **DOR**  **(LR+ / LR-)** | ***X^2^*** | ***Kappa value*** | ***P* value** |
| --- | --- | --- | --- | --- | --- | --- | --- | --- | --- | --- | --- | --- | --- | --- | --- |
| **CONS1** | **Nested A + AN** | 100 | 50 | 98.4 | 100 | 50.0 | 0.0 | 98.4 | 75 | 2.00 | 0.00 | - | 7.120 | 0.659 | 0.01 |
|  | **Hcom1** | 100 | 50 | 98.4 | 100 | 50 | 0.0 | 98.4 | 75 | 2.0 | 0.0 | - | 7.120 | 0.659 | 0.01 |
|  | **H276** | 98.3 | 50 | 98.3 | 50.0 | 50.0 | 1.7 | 96.8 | 74.2 | 1.97 | 0.033 | 59 | 3.140 | 0.483 | 0.076 |
|  | **Outer + Inner** | 100 | 100 | 100 | 100 | 0.0 | 0.0 | 100 | 100 | - | 0.0 | - | 34.10 | 1.00 | 0.0001 |
|  | **Heli-nestS + Heli** | 8.3 | 50 | 83.3 | 1.8 | 50 | 91.7 | 9.7 | 29.2 | 0.17 | 1.83 | 0.09 | 0.555 | -0.030 | 0.456 |

SEN: sensitivity; SPC: specificity; PPV: Positive predictive value; NPN: Negative predictive value; FPR: False positive rate; FNR: False negative rate; ACC: Accuracy; BA: Balanced accuracy; LR+: Positive likelihood ratio; LR-: Negative likelihood ratio; DOR: Diagnostic odds ratio; *X^2^*: Chi-Square (Continuity Correction).

16S rRNA, hspG and Heid primers tested negative in detection of all 62 *Helicobacter* species (in silico two mismatch). 16S rRNA-F and BFHpyl primers tested negative in detection of 61 *Helicobacter* species (in silico two mismatch).

**Table 4.** **Results of screening primers in detection of *Helicobacter* species (8 sequenced DNA) and** **Non-*Helicobacter* species (10 sequenced DNA)**

| **Screening Primers** | ***Helicobacter* spp. (n = 8)** | | **Non-*Helicobacter* spp. (n= 10)** | |
| --- | --- | --- | --- | --- |
|  | **Positive** | **Negative** | **Positive** | **Negative** |
| **CONS1** | 8 (100%) | 0 (0%) | 0 (0%) | 10 (100%) |
| **Outer + Inner** | 8 (100%) | 0 (0%) | 10 (100%) | 0 (0%) |
| **H276** | 8 (100%) | 0 (0%) | 1 (10%) | 9 (90%) |
| **16 XF** | 8 (100%) | 0 (0%) | 1 (10%) | 9 (90%) |
| **16S rRNA** | 6 (75%) | 2 (25%) | 0 (0%) | 10 (100%) |
| **Heli-nestS + Heli** | 8 (100%) | 0 (0%) | 1 (10%) | 9 (90%) |
| **Hcom1** | 8 (100%) | 0 (0%) | 1 (10%) | 9 (90%) |
| **BFHpyl** | 1 (12.5%) | 7 (87.5%) | 1 (10%) | 9 (90%) |
| **Heid** | 0 (0%) | 8 (100%) | 1 (10%) | 9 (90%) |

**Table 5.** **Results of screening primers in detection of *Helicobacter pylori* (5 sequenced DNA)**

| **Screening Primers** | ***Helicobacter pylori* (n = 5)** | |
| --- | --- | --- |
|  | **Positive** | **Negative** |
| **CONS1** | 5 (100%) | 0 (0%) |
| **Nested A + AN** | 5 (100%) | 0 (0%) |

**Table 5. Screening primers results for detection of *Helicobacter* spp. (n= 8) and Non-*Helicobacter* spp. (n= 10)**

| **Gold standard Primer** | **Screening Primer** | **SEN (%)** | **SPC (%)** | **PPV (%)** | **NPV (%)** | **FPR (%)** | **FNR (%)** | **Acc (%)** | **BA (%)** | **LR+** | **LR-** | **DOR**  **(LR+ / LR-)** | ***X^2^*** | ***Kappa* value** | ***P* value** |
| --- | --- | --- | --- | --- | --- | --- | --- | --- | --- | --- | --- | --- | --- | --- | --- |
| **CONS1** | **H276** | 100 | 90.0 | 88.9 | 100.0 | 10.0 | 0.0 | 94.4 | 95 | 10.0 | 0.0 | - | 11.025 | 0.889 | 0.001 |
|  | **16 XF** | 100 | 90.0 | 88.9 | 100.0 | 10.0 | 0.0 | 94.4 | 95 | 10.0 | 0.0 | - | 11.025 | 0.889 | 0.001 |
|  | **Heli-nestS + Heli** | 100 | 90.0 | 88.9 | 100.0 | 10.0 | 0.0 | 94.4 | 95 | 10.0 | 0.0 | - | 11.025 | 0.889 | 0.001 |
|  | **Hcom1** | 100 | 90.0 | 88.9 | 100.0 | 10.0 | 0.0 | 94.4 | 95 | 10.0 | 0.0 | - | 11.025 | 0.889 | 0.001 |
|  | **16S rRNA** | 75.0 | 100 | 100 | 83.3 | 0.0 | 25 | 88.9 | 87.5 | - | 0.25 | - | 8.128 | 0.769 | 0.004 |
|  |  |  |  |  |  |  |  |  |  |  |  |  |  |  |  |
|  | **BFHpyl** | 12.5 | 90 | 50 | 56.3 | 10 | 87.5 | 55.6 | 51.3 | 1.25 | 0.97 | 1.29 | 0.001 | 0.27 | 1 |
|  | **Heid** | 0.0 | 90 | 0.0 | 52.9 | 10 | 100 | 50.0 | 45 | 0.0 | 1.11 | 0.0 | 0.001 | -0.110 | 1 |

SEN: sensitivity; SPC: specificity; PPV: Positive predictive value; NPN: Negative predictive value; FPR: False positive rate; FNR: False negative rate; ACC: Accuracy; BA: Balanced accuracy; LR+: Positive likelihood ratio; LR-: Negative likelihood ratio; DOR: Diagnostic odds ratio.

The Receiver Operating Characteristics ROC analysis plots TRP (Sensitivity) *vs* FPR (1-specificity). The area under the curve (AUC) is a parameter indicating the intrinsic accuracy of the diagnostic test in diagnosis of *Helicobacter* species. The larger area (AUC), the more accurate the diagnostic test. The accuracy of diagnostic test was classified into excellent, good, worthless and not good based on the Area Under Curve (AUC). When the AUC was less than 0.7, the diagnostic test was classified as not reliable test, whereas the diagnostic test was considered reliable and accurate at AUC more than 0.8.


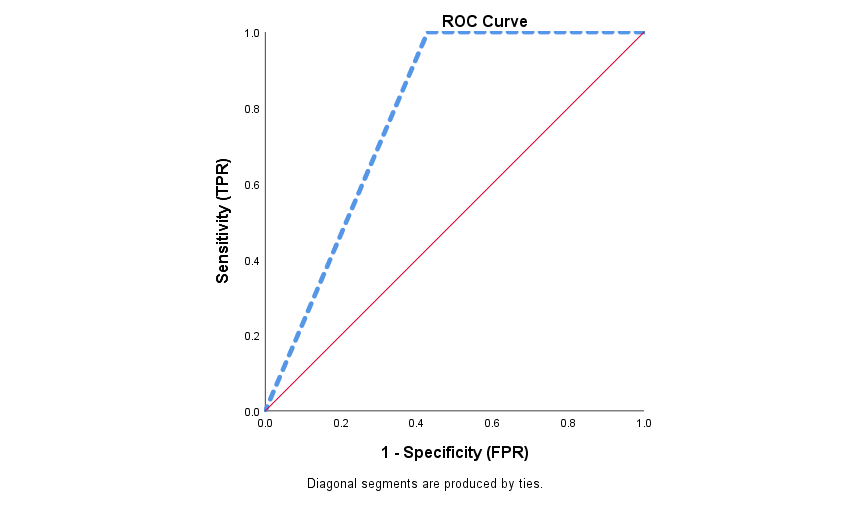


**Figure (1): ROC curve of CONS1 and Nested A + AN primer for detection of 62 Helicobacter strains (in silico no mismatch)**. Null hypothesis: true area = 0.5, AUC: Area under the curve, AUC represents accuracy of screening test (0.786).


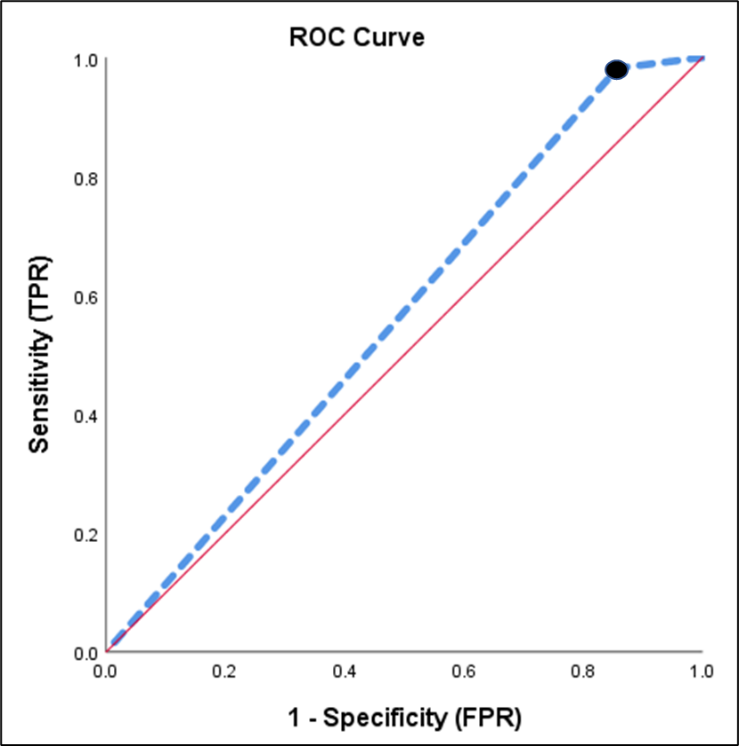


**Figure (2): ROC curve of CONS1 and H276 primers for detection of 62 Helicobacter strains (in silico no mismatch)**. Null hypothesis: true area = 0.5, AUC: Area under the curve, AUC represents accuracy of screening test (0.562).


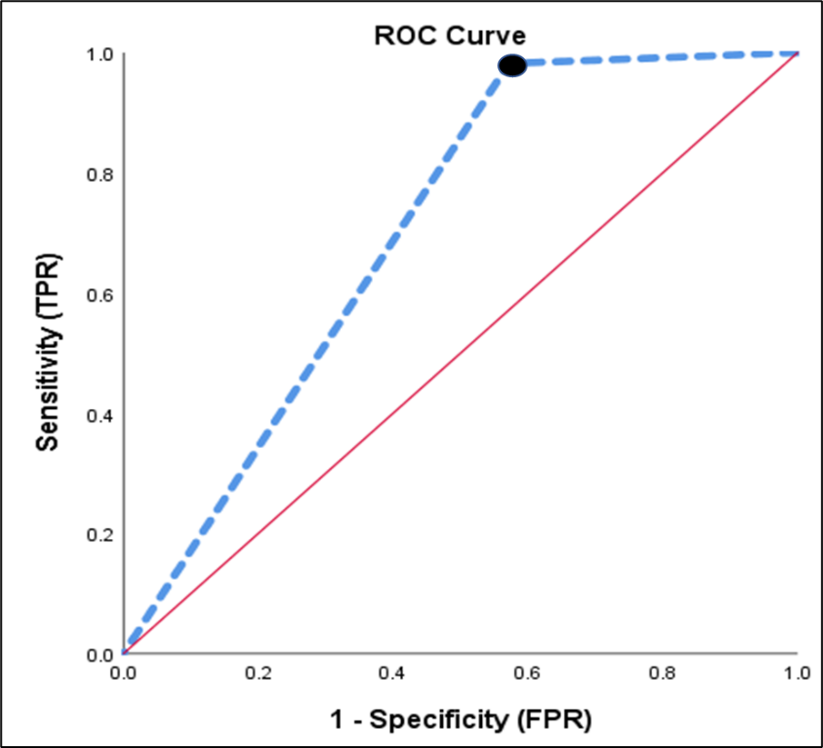


**Figure (3): ROC curve of CONS1 and Outer + Inner primers for detection of 62 Helicobacter strains (in silico no mismatch).** Null hypothesis: true area = 0.5, AUC: Area under the curve, AUC represents accuracy of screening test (0.705).


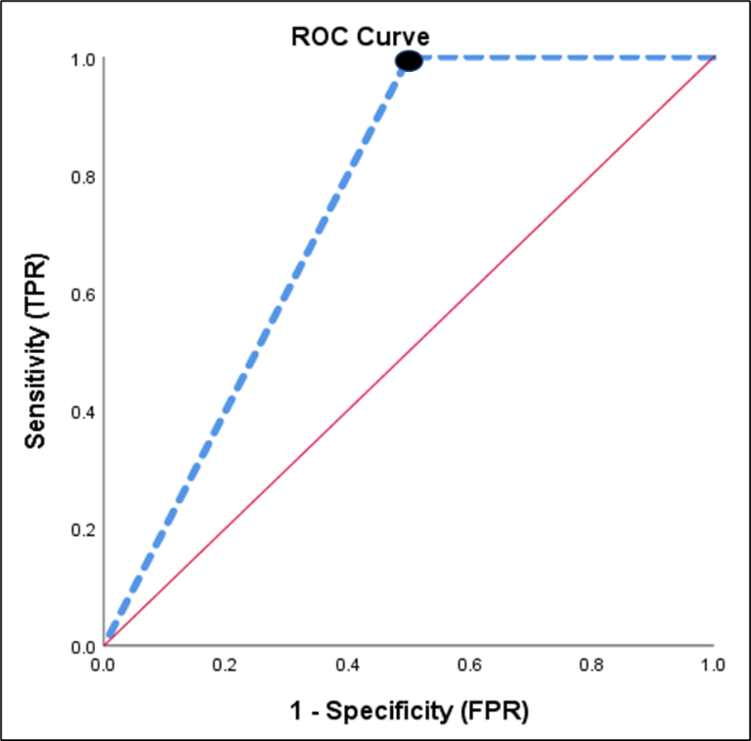


**Figure (4): ROC curve of CONS1 and Nested A + AN primer for detection of 62 Helicobacter strains (in silico two mismatch)**. Null hypothesis: true area = 0.5, AUC: Area under the curve, AUC represents accuracy of screening test (0.750).


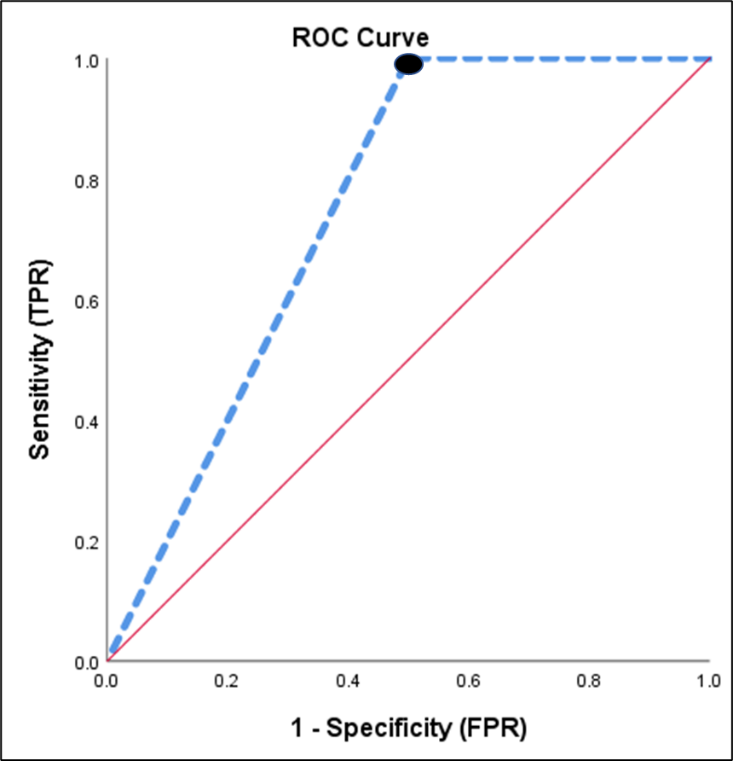


**Figure (5): ROC curve of CONS1 and Hcom1 primers for detection of 62 Helicobacter strains (in silico two mismatch)**. Null hypothesis: true area = 0.5, AUC: Area under the curve, AUC represents accuracy of screening test (0.750).


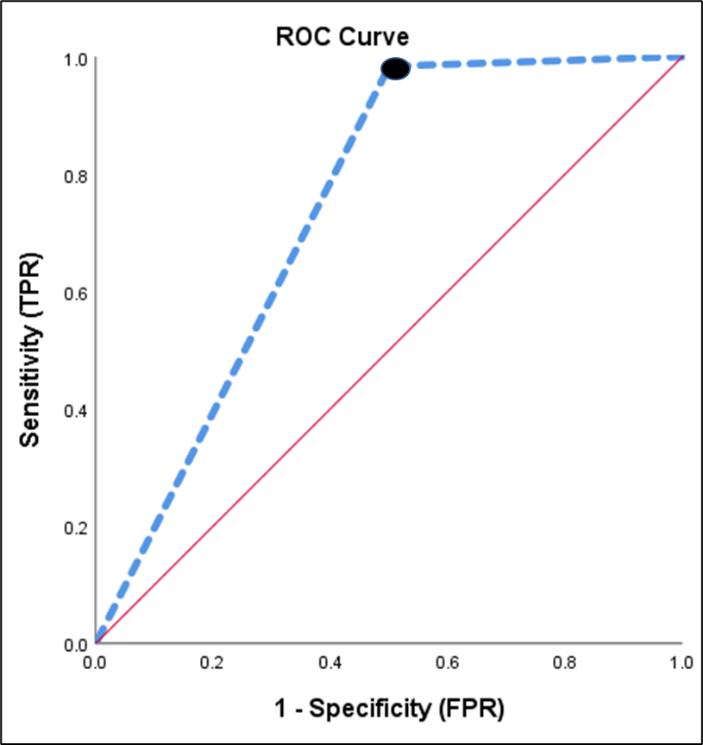


**Figure (6): ROC curve of CONS1 and H276 primers for detection of 62 Helicobacter strains (in silico two mismatch)**. Null hypothesis: true area = 0.5, AUC: Area under the curve, AUC represents accuracy of screening test (0.742).


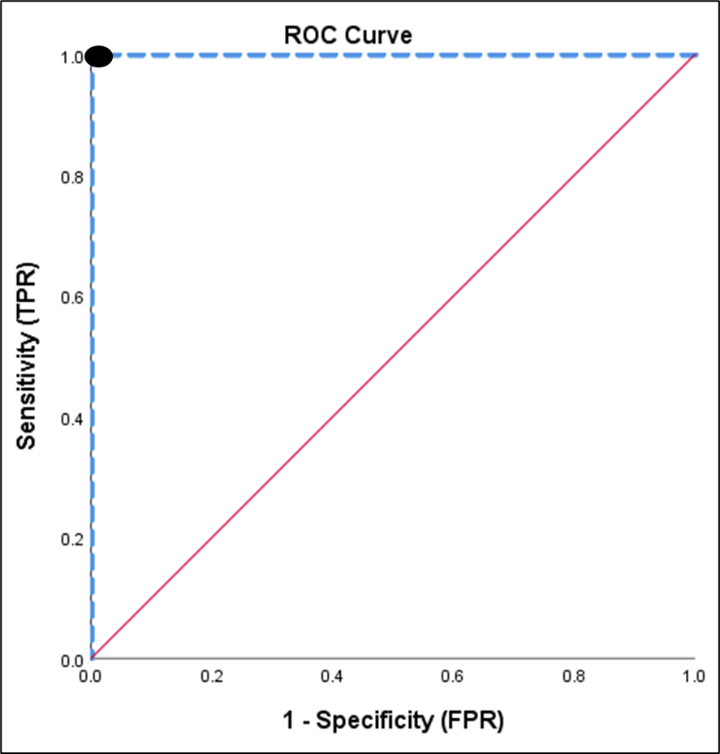


**Figure (7): ROC curve of CONS1 and Outer + Inner primers for detection of 62 Helicobacter strains (****in silico two mismatch)**. Null hypothesis: true area = 0.5, AUC: Area under the curve, AUC represents accuracy of screening test (1.00).


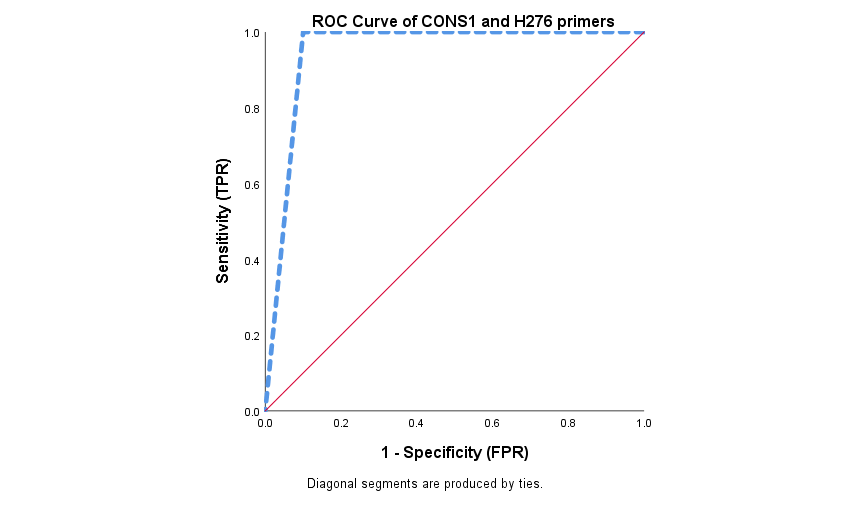


**Figure (8): ROC curve of CONS1 and H276 primers for detection of *Helicobacter* spp. (n= 8) and Non-*Helicobacter* spp. (n= 10).** Null hypothesis: true area = 0.5, AUC: Area under the curve, AUC represents accuracy of screening test (0.950).


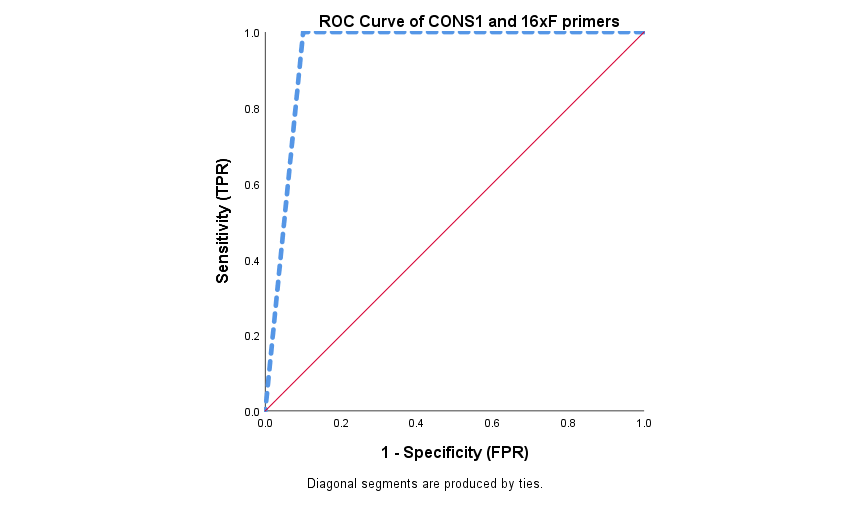


**Figure (9): ROC curve of CONS1 and 16xF primers for detection of *Helicobacter* spp. (n= 8) and Non-*Helicobacter* spp. (n= 10).** Null hypothesis: true area = 0.5, AUC: Area under the curve, AUC represents accuracy of screening test (0.950).


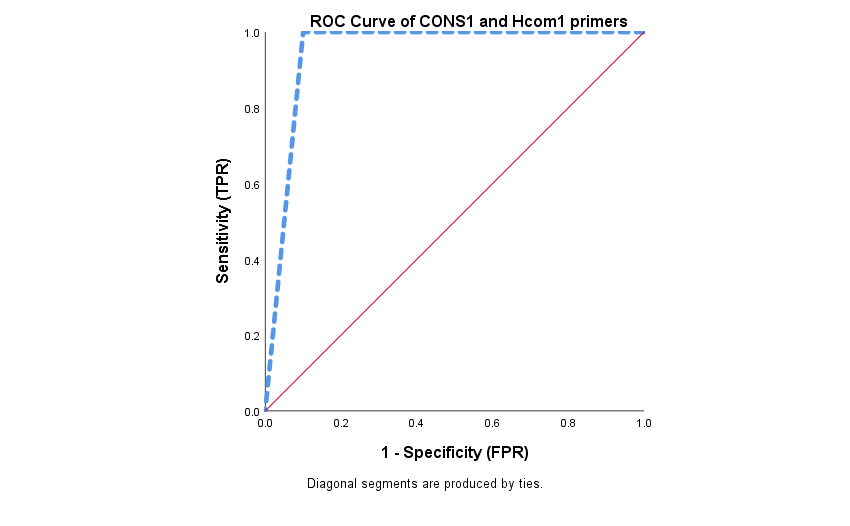


**Figure (10): ROC curve of CONS1 and Hcom1 primers for detection of *Helicobacter* spp. (n= 8) and Non-*Helicobacter* spp. (n= 10**). Null hypothesis: true area = 0.5, AUC: Area under the curve, AUC represents accuracy of screening test (0.950).


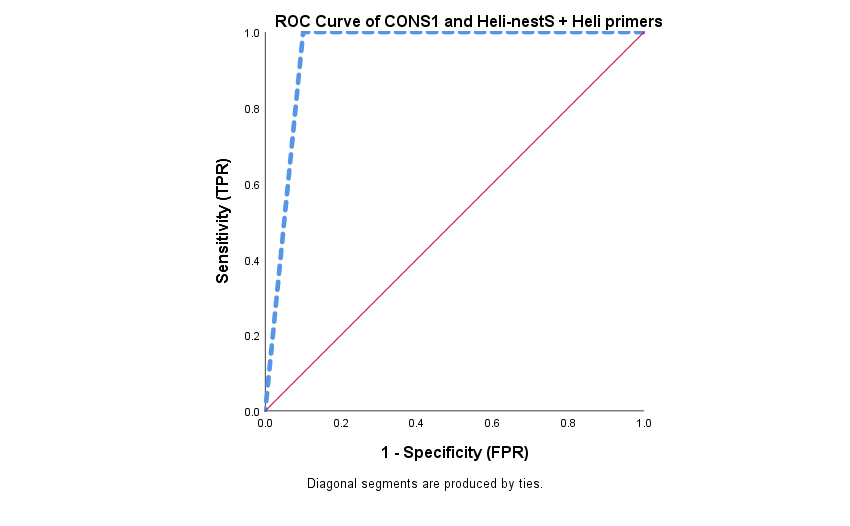


**Figure (11): ROC curve of CONS1 and Heli-nestS + Heli primers for detection of *Helicobacter* spp. (n= 8) and Non-*Helicobacter* spp. (n= 10).** Null hypothesis: true area = 0.5, AUC: Area under the curve, AUC represents accuracy of screening test (0.950).


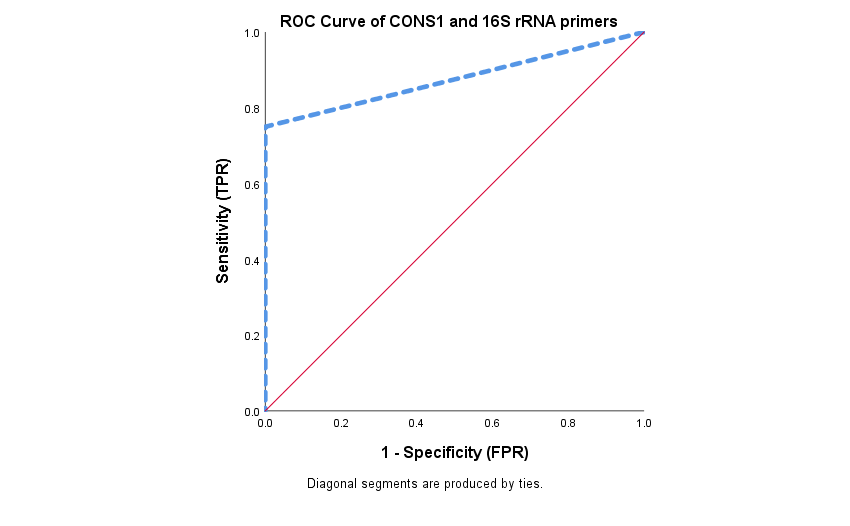


**Figure (12): ROC curve of CONS1 and 16S rRNA primers for detection of *Helicobacter* spp. (n= 8) and Non-*Helicobacter* spp. (n= 10).** Null hypothesis: true area = 0.5, AUC: Area under the curve, AUC represents accuracy of screening test (0.875).


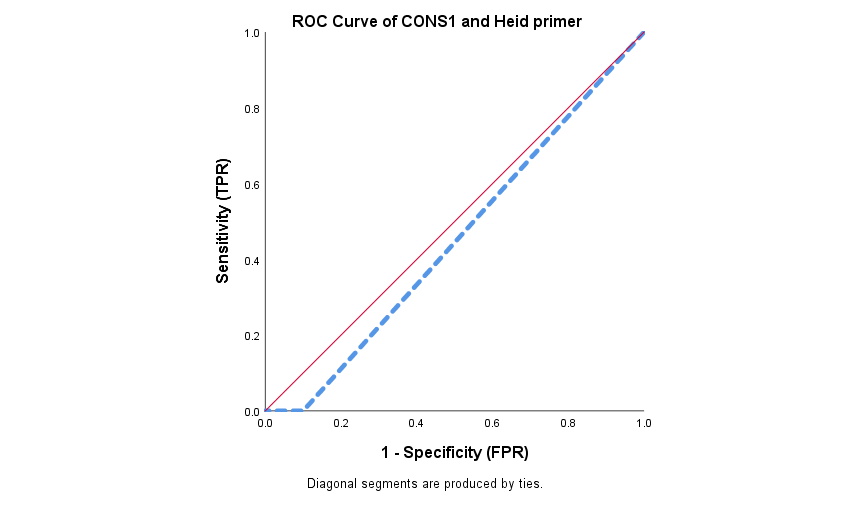


**Figure (13): ROC curve of CONS1 and Heid primers for detection of *Helicobacter* species (n= 8) and Non-*Helicobacter* spp. (n= 10).** Null hypothesis: true area = 0.5, AUC: Area under the curve, AUC represents accuracy of screening test (0.450).


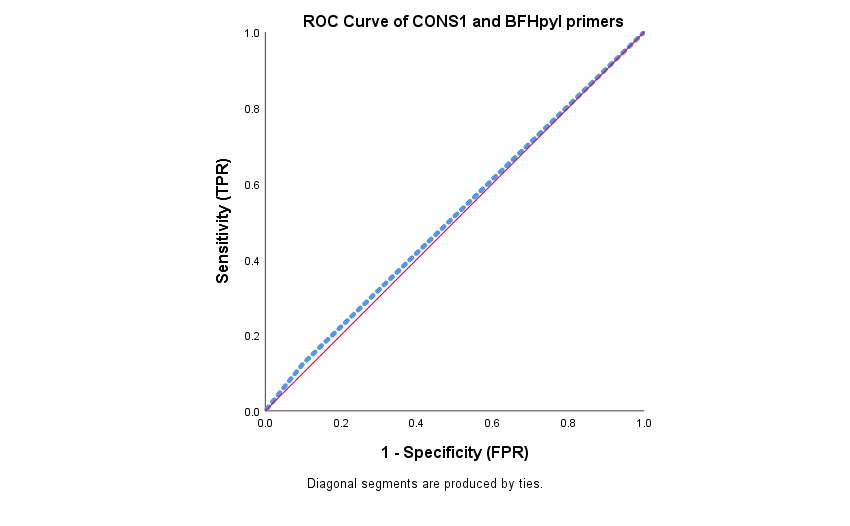


**Figure (14): ROC curve of CONS1 and BFHpyl primers for detection of *Helicobacter* species (n= 8) and Non-*Helicobacter* spp. (n= 10).** Null hypothesis: true area = 0.5, AUC: Area under the curve, AUC represents accuracy of screening test (0.513).
