# Supplementary material for: The validity evaluation of different 16srRNA gene primers for helicobacter detection urgently requesting to design new specific primers
Source: Sci Rep. 2022 Jun 24;12:10737. doi: 10.1038/s41598-022-14600-4 (PMC9232570; doi:10.1038/s41598-022-14600-4)
Supplement: Supplementary file 7 — Supplementary Information 7. [file 41598_2022_14600_MOESM7_ESM.docx]

**Supplementary figures legends**

Figure 1 . Gel electrophoresis of PCR products of ConsH amplified products(435bp) in reference to 100 bp DNA ladder.

Figure 2. Gel electrophoresis of PCR products of PyloA amplified products (1274bp) in reference to 100 bp DNA ladder.

Figure 3. Gel electrophoresis of PCR products of PyloAN amplified products (160 bp) in reference to 100 bp DNA ladder.

Figure 4. A, Sensitivity testing of primers ConsH and C, Sensitivity testing of PyloA using Stool clinical sample DNA with different concentrations. B, Sensitivity testing of Primers ConsH and D, Sensitivity testing of PyloA using biopsy clinical sample DNA with different concentrations.
